# Supplementary material for: Gestational Pesticide Exposure and Child Respiratory Health
Source: Int J Environ Res Public Health. 2020 Sep 30;17(19):7165. doi: 10.3390/ijerph17197165 (PMC7579149; doi:10.3390/ijerph17197165)
Supplement: Supplementary file 1 [file ijerph-17-07165-s001.pdf]

Supplementary Materials

Table S1. Odds ratios and p values from logistic regression analyses examining contributions of maternal pesticide metabolite concentration during pregnancy and the interactions of these exposures with maternal daily fresh fruit and vegetable consumption, and *PON1-108* and *PON1192* alleles to child wheeze.

	**Direct**		***Fr/Veg*** *(ref <daily)*		***PON1-108*** *(ref QR/RR)*		***PON1192*** *(ref CC)*			
							CT		***TT***	
Wheeze	**OR**	***p***	**OR**	***p***	**OR**	***p***	**OR**	***p***	**OR**	***p***
Wheeze age 8 years†										
DE	1.25	0.108	1.30	0.710	0.43	0.005	0.84	0.510	2.94	0.096
DM	1.06	0.652	1.22	0.495	0.50	0.079	0.58	0.046	2,08	0.181
DAP	1.13	0.402	1.21	0.542	0.40	0.036	0.53	0.057	2.81	0.091
3PBA	0.99	0.918	0.74	0.038	0.898	0.652	0.84	0.310	0.64	0.078
Any wheeze to 8 years‡										
DE	0.99	0.394	0.98	0.362	0.86	0.138	0.85	0.547	0.50	0.749
DM	1.00	0.324	0.96	0.159	0.86	0.134	0.90	0.337	1.10	0.638
DAP	0.92	0.284	1.22	0.213	0.87	0.405	1.05	0.755	1.50	0.129
3PBA	0.99	0.800	0.74	0.007	0.85	0.198	0.82	0.118	0.73	0.141

DE = diethyl phosphate, DM = dimethyl phosphate, DAP = dialkyl phosphate, 3PBA = pyrethroid (3-Phenoxybenzoic acid); Direct analyses include no covariates.

Covariates in the logistic regression models, except the direct models, predicting wheeze at age 8 years were child's birth weight and sex, mother's age at delivery, employment, education, alcohol consumption, parity, and daily fruit and vegetable consumption.

Covariates in the generalized linear mixed models, except the direct models, predicting any wheeze at ages to 8 years were child's birth weight and sex, and mother's age at delivery, education, employment, parity, alcohol consumption and daily fruit and vegetable consumption during pregnancy.

Table 2. Correlations of maternal pesticide metabolite concentration with predicted FEV1 at 4 (n = 155) and 5 years (n = 197).

		**DAP**	**DE**	**DM**	**3PBA**
Percent Predicted FEV1 4 Years	Correlation	0.21	0.004	−0.008	−0.06
	p	(0.008)	(0.9615)	(0.92)	(0.49)
Percent Predicted FEV1 5 Years	Correlation	-0.02	−0.008	0.03	0.03
	p	(0.83)	(0.91)	(0.66)	(0.66)

DAP=dialkyl phosphate, DE=diethyl phosphate, DM=dimethyl phosphate, 3PBA = pyrethroid.
